# Colour Counts: Sunlight and Skin Type as Drivers of Vitamin D Deficiency at UK Latitudes

**DOI:** 10.3390/nu10040457

**Published:** 2018-04-07

**Authors:** Ann R. Webb, Andreas Kazantzidis, Richard C. Kift, Mark D. Farrar, Jack Wilkinson, Lesley E. Rhodes

**Affiliations:** 1 School of Earth and Environmental Sciences, Faculty of Science and Engineering, University of Manchester, Manchester, M13 9PL, UK; ann.webb@manchester.ac.uk (A.R.W.); akaza@upatras.gr (A.K.); richard.kift@manchester.ac.uk (R.C.K.); 2 Physics Department, University of Patras, 26500 Patras, Greece; 3 Faculty of Biology, Medicine and Health, University of Manchester and Dermatology Centre, Salford Royal NHS Foundation Trust, Manchester Academic Health Science Centre, Manchester, M6 8HD UK; mark.farrar@manchester.ac.uk; 4 Centre for Biostatistics, School of Health Sciences, Faculty of Biology, Medicine and Health, Manchester Academic Health Science Centre (MAHSC), University of Manchester, Manchester, M13 9PL UK; jack.wilkinson@manchester.ac.uk

**Keywords:** vitamin D, ultraviolet radiation, climatology, skin type V, dietary intake, vitamin D deficiency

## Abstract

Sunlight exposure, with resulting cutaneous synthesis, is a major source of vitamin D for many, while dietary intake is low in modern diets. The constitutive pigment in skin determines skin type, observed as white, brown, or black skin. The melanin pigment absorbs ultraviolet radiation (UVR) and protects underlying skin from damage caused by UVR. It also reduces the UVR available for vitamin D synthesis in the skin. It has been shown that the white-skinned population of the UK are able to meet their vitamin D needs with short, daily lunchtime exposures to sunlight. We have followed the same methodology, based on a 10-year UK all-weather UVR climatology, observation (sun exposure, diet, vitamin D status), and UVR intervention studies with Fitzpatrick skin type V (brown) adults, to determine whether sunlight at UK latitudes could provide an adequate source of vitamin D for this section of the population. Results show that to meet vitamin D requirements, skin type V individuals in the UK need ~25 min daily sunlight at lunchtime, from March to September. This makes several assumptions, including that forearms and lower legs are exposed June–August; only exposing hands and face at this time is inadequate. For practical and cultural reasons, enhanced oral intake of vitamin D should be considered for this population.

## 1. Introduction

Vitamin D, necessary for musculoskeletal health, and potentially advantageous for prevention of a range of other diseases [1] is a unique nutrient in that the body can synthesise its own vitamin D. It does this via the action of ultraviolet radiation (UVR) on 7-dehydrocholesterol in skin, eventually leading to vitamin D, and then the main circulating form 25 hydroxyvitamin D (25(OH)D), used as a measure of vitamin D status), and the active metabolite 1,25 dihydroxyvitamin D, which is closely regulated by the endocrine system [2]. Vitamin D is also available through the diet, though it is naturally present in more than low quantity in few foods (fatty fish are the major source). Modern western-style diets are unlikely to meet the recommended dietary intake of 10 μg/day [3] advised both in the USA [4] and recently in the UK [5]. This leaves cutaneous synthesis as the primary source of vitamin D for many, but the process is determined by a complex range of variables, predominantly available UVB (weather/climate), exposure time and pattern, skin area exposed, skin pigment, and age [6].

When considering different sub-populations resident in a given location, the weather and climate are the same for all. It is personal characteristics and behavior that determine how much vitamin D can be made through sun exposure, and hence the vitamin D status of the sub-populations. Skin pigmentation, i.e., melanin, absorbs the UVR that initiates vitamin D synthesis, and hence decreases the vitamin D that is made for a given exposure compared to less pigmented skin. This has been observed in UVR intervention studies [7] and more generally. Recent analysis of the UK-based National Diet and Nutrition Survey (NDNS)—4 year Rolling Programme showed that the prevalence of serum 25(OH)D < 30 nmol/L in Asian participants (*n* = 52) was 59.6%, compared to 19.6% in white participants (*n* = 1359), with similar differences between pigmented and non-pigmented skin types in other European countries at similar or higher latitudes [8]. Improving the vitamin D status of the skin type V population requires either an oral intervention through food fortification or vitamin D supplements, or a change in behavior to increase the vitamin D synthesized in the skin. Much of the public health guidance available for sun exposure encourages sun protection, taking little account of skin type, while cultural expectations may limit skin exposure. Thus, the UK National Institute for Health and Care Excellence (NICE) has recently identified the need for targeted sun exposure advice for different sections of the population [9], but the guidance in terms of required sun exposure for vitamin D synthesis has not previously been quantified for skin type V individuals. Here we present that calculation, based on previous in vivo studies of skin type V adults [7,10,11] and a high resolution UVR climatology of the UK [12]. Following similar work for a white-skinned population [13], this allows for the targeted advice called for by NICE.

## 2. Materials and Methods 

The methods of calculating the sunlight exposure required to maintain a vitamin D status above deficiency (≥25 nmol/L circulating 25(OH)D) year-round (expressed as minutes unprotected skin is exposed to sunlight in the summer months) are described in detail for white Caucasians [13] and are followed here for skin type V individuals. 

In summary, the ambient UVR across the UK was calculated for each day of a 10-year period using satellite inputs of ozone, cloud, aerosol optical depth, and accounting for altitude, to produce a 10-year all-weather climatology [12]. UVB irradiance at the Earth’s surface is so low at this latitude in the winter months that no appreciable vitamin D can be made in skin, so summer sunlight exposure has to provide adequate vitamin D to remain above the target status until the next spring. Datasets from previously published work conducted in Greater Manchester (53.5° N) provided the observed UVR exposures, dietary intake and vitamin D status of skin type I-IV [14] and skin type V individuals [10] year-round enabling an estimate of the end-summer circulating 25(OH)D needed to remain at or above 25 nmol/L throughout the winter, and the monthly spend of 25(OH)D by the body. Then, the response of skin type V adults to an intervention of known doses of simulated sunlight [11] was used to calculate the UVR dose required to raise circulating 25(OH)D from the winter-end low to the summer-end high previously assessed. This assumes that, like the intervention study, the UVR is received in small doses on a regular basis. A safe exposure time for skin type V was estimated based on the highest expected UVR irradiance in the UK and then the UV climatology was used to assess whether daily noon-time exposures, of duration the safe exposure time, throughout the summer months, would provide for the target end-summer 25(OH)D under the UV climate of the UK. 

The vitamin D made in skin and quantified in the circulating 25(OH)D also depends on skin area exposed. Thus, calculations were repeated for four different scenarios (S1–4) of skin area exposed: S1, 35% (hands, face, forearms, and lower legs) March–September; S2, 10% (hands and face) March–May and September but 35% June–August; S3, 10% March–September; S4, 35% June–August only, but with exposure time adjusted by latitude to give the same dose anywhere in the UK. The intervention studies [7,11] were conducted with 35% skin area exposed, although over a shorter period of 6 weeks, so scenarios S1 and S4 relate directly to the underlying in vivo study. Where the model assumes 10% skin area exposed the results were scaled by skin area i.e., it was assumed that all skin makes vitamin D in an equivalent way when exposed to UVB radiation. Furthermore, no account was taken of possible differences in skin photohardening with repeated UVR exposure over 6 months rather than 6 weeks. The ambient (modelled) UVR was also adjusted from radiation on a horizontal surface (base climatology) to radiation on a randomly oriented vertical surface, according to [15], to be more representative of an upright human body. The base climatology (horizontal surface) was retained in assessing the safe exposure time, as a safeguard for horizontal areas of the upright body (e.g., shoulders) or for those who may sit or lie while outdoors. Thus, the model conditions err on the side of caution with respect to sunburn and may underestimate the vitamin D that can be synthesized if behaviour is other than upright standing or walking.

The results were then used to determine a simple public health message on vitamin D acquisition and the maintenance of sufficient vitamin D status, for skin type V individuals.

## 3. Results

### 3.1. Input to the Vitamin D Synthesis Calculations

**Figure 1:**
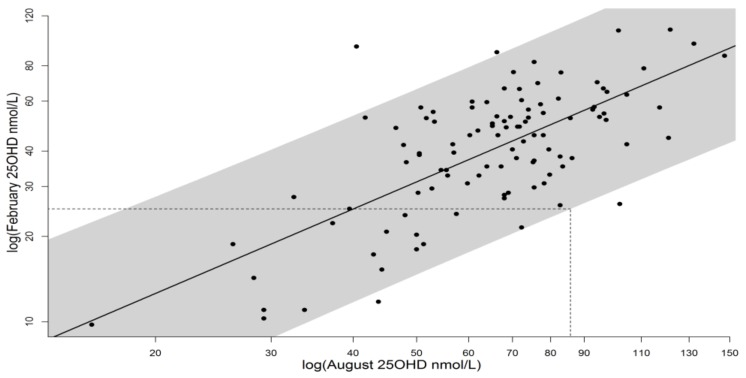
Log-log regression of February 25(OH)D on August 25(OH)D, as described in [13], data from [10,14]. Data points are individual volunteers, shaded band shows 96% prediction interval, under which 97.5% of individuals would exceed 25 nmol/L in February given an August level of 85.8 nmol/L. White Caucasian vitamin D spend data have been used as a proxy for South Asian vitamin D spend as explained in Section 3.1.

The observation study [10] showed that the dietary intake of the skin type V population was very low (median 1.32 µg per day). It also showed a slight seasonal cycle in both UVR exposure and circulating 25(OH)D, indicating that sunlight exposure does contribute to vitamin D status for people with skin type V in UK. However, UVR exposure, the amplitude of the seasonal change in 25(OH)D, and the absolute levels of 25(OH)D were all less than for white Caucasians [16]. Median values of circulating 25(OH)D were 22.5 nmol/L in summer and 14.5 nmol/L in winter, while 93% of the cohort remained below 50 nmol/L year-round, defined as insufficiency [4]. Given that many of this group had circulating 25(OH)D < 25 nmol/L all year round, it was not possible to regress winter on summer values to determine what end-summer value of 25(OH)D would result in an end-winter value of ≥25 nmol/L, nor to determine monthly spend of 25(OH)D in this way. Therefore we used the calculations previously performed for white Caucasians [13], Figure 1, as a proxy for vitamin D spend in the south Asian (skin type V) group, on the assumption that if 25(OH)D status could be boosted once (by supplements or UVR) to levels of the white Caucasian population then from the point that vitamin D enters the bloodstream its metabolism will be the same, irrespective of external skin colour. There was no suggestion of differences in rates of spending between white and skin type V cohorts, based on a statistical interaction test. Further, for the few skin type V individuals who reached relatively high 25(OH)D levels the data fit well with the white Caucasian regression. For the white Caucasian calculations [13] the period of the year with no significant vitamin D synthesis was taken as October to February inclusive, based on observational study [14]. Due to UVR absorption by skin pigment (melanin), a shorter summer synthesis period was used for skin type V and the end-summer target identified in August (as given in Table 1), while in the final calculation of sun exposure required, extra time of spend-only had to be accounted for.

The intervention study [11] showed that skin type V needs a dose of simulated sunlight that is 2.5–3 times that required by white Caucasians to raise circulating 25(OH)D by the same amount over a 6 week period. For this reason, the dose for which the ‘safe exposure time’ for skin type V was set was 2.75 SED, which according to reported MED for people of different skin types is also well below the erythema threshold for skin type V [17]. The ‘safe exposure time’, that is the time to achieve 2.75 SED on the UK’s south coast at noon on a clear day in June, was 25 min. Table 1 summarises the intermediate results (model parameters) and the possibility of maintaining a 25(OH)D level ≥ 25 nmol/L year-round under each exposure scenario and the UK climatology, based on those parameters.

### 3.2. Application for UK Climatology

**Figure 2:**
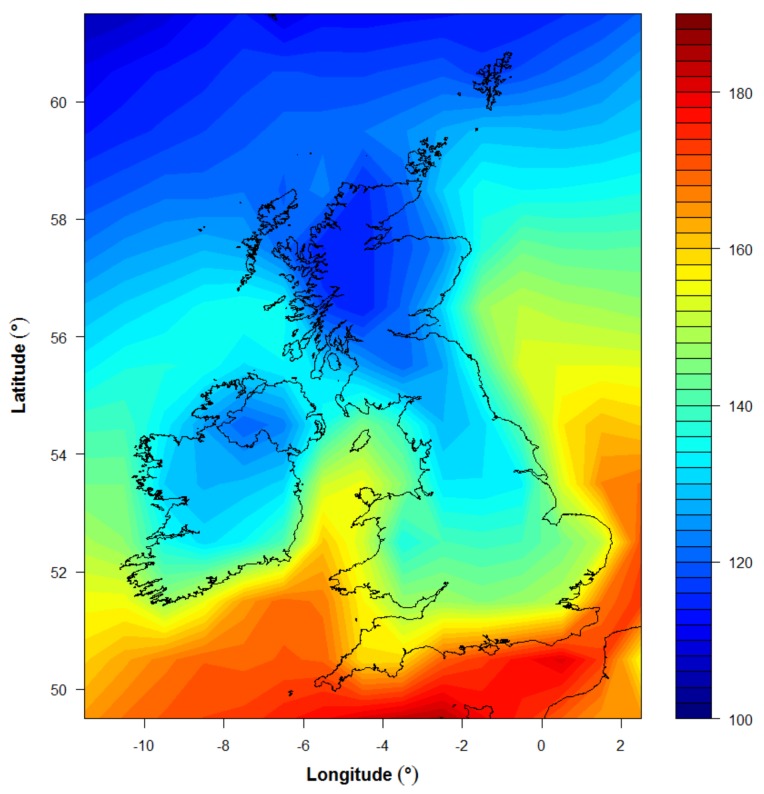
Total summer half year (March–September) exposure on a randomly oriented vertical surface for a daily 25 min exposure at lunchtime according to Scenario S2 (10% skin surface area exposed March–May and September, 35% June–August). Exposures in March–May and September were scaled by skin area exposed before being included in the total, to indicate the reduced capacity for vitamin D synthesis associated with the reduced skin area exposed. The colour scale illustrates erythema effective UV (Jm^−2^), for comparison with the summer target value of 89.6 SED from Table 1.

Figure 2 illustrates the calculation of achievable SED across the UK for 25 min daily exposure of skin type V skin, adjusted to a vertical surface, for comparison with the target summer dose required (89.6 SED or 8960 Jm^−2^ : see Table 1). The calculation shown is for scenario S2: 10% skin area (hands and face) exposed from March–May and September, plus 35% skin area exposed (hands, face, forearms, and lower legs) June–August. For this scenario, and by extension for scenario S1 (35% skin area exposed March–September), it appears possible to meet vitamin D requirements through sun exposure for a skin type V individual living in the UK. However, the commitment to daily noon-time sun exposure March–September, at 25 min duration, is significantly more than required by a white Caucasian individual (9 min [12]).

The results for scenarios S3 and S4 are provided in Table 1. Exposing hands and face only throughout the summer (S3), for the 25 min skin type V exposure time, is inadequate to provide for maintenance of vitamin D status throughout the winter months. When exposure is only specified for the months June–August (35% skin area) but the exposure time adjusted by latitude to provide 2.75 SED across the UK (S4), it is possible to maintain year-round vitamin D status by means of this daily sun exposure. However, in this scenario the exposure time becomes as much as 40 min at lunchtime every day, requiring significant commitment of both time and dress (skin area exposed) for these 3 months, particularly at more northern latitudes.

## 4. Discussion

Skin type V individuals have, by definition, significant constitutive pigmentation that gives their skin its brown colour. The melanin pigment protects the underlying skin against damage from UVR. In reducing the UVR in this way it also reduces the vitamin D synthesis due to the same UVR. Thus, theory suggests, and our in vivo studies have shown [7,16] that skin type V individuals need more UVR (achieved naturally by more sun exposure) to make the same amount of vitamin D in skin as white Caucasians who lack their constitutive pigmentation. At mid-high latitudes where the climate is relatively UVR-poor (e.g., the UK) this can put the skin type V population at greater risk of vitamin D deficiency than their white-skinned counterparts. This was demonstrably true in our earlier in vivo observation [10] and intervention [7] studies, and in conversation with representatives of the South Asian population in Manchester [18].

The low levels of circulating 25(OH)D observed during the underlying in vivo studies means that several assumptions have been made in the current calculations, including the ability to reach 25(OH)D levels of ≥85.8 nmol/L (end summer target), which was not observed in UK South Asians in daily life [10] nor following intervention with a range of doses of simulated sunlight, whilst wearing summer clothing to reveal 35% skin surface area [11]. This lack of high levels of circulating 25(OH)D also led to assumptions that spend of 25(OH)D is independent of skin type and a similar summer-winter regression can be used in determining the end-summer level of circulating 25(OH)D required to remain ≥25 nmol/L throughout the winter. 

Depending on the selected sun exposure scenario, the daily lunchtime exposure for those of skin type V varies from 25 to 40 min across the UK during the summer months and involves at least 35% skin area exposure for the period June–August. This is a significant practical commitment. It should be emphasized that these scenarios involve sunlight exposure of unprotected skin, i.e., skin to which sunscreen has not been applied, as sunscreen use would prolong the time required, dependent on the adequacy of its application [19]. Additionally, facial products such as moisturizers and foundation often contain sunscreen agents, thus providing a sun protection factor (SPF) to this site. Whilst our observation study of South Asian skin type V individuals in the UK [10] found infrequent use of dedicated sunscreen products or SPF-containing facial products in this community, this may not be universal, and could change. Moreover, there is controversy concerning the level of circulating 25(OH)D that should be maintained. Whilst the UK authorities recommend maintaining a year-round level of ≥25 nmol/L, i.e., above vitamin D deficiency status [5], others including the USA/Canadian and European authorities recommend maintaining a level of ≥50 nmol/L, i.e., sufficiency status [4,20].

Increasing skin area exposed above 35% might further improve 25(OH)D levels, and/or reduce exposure time required to maintain ≥25 nmol/L in the UK and could also help minimize the impact of the increased skin photoadaptation occurring over a longer period of UVR exposures. However, it could be impractical for many, and some South Asians have cultural reasons for exposing no more than hands and face in public. As for white Caucasians [12], this latter scenario (S3) of only hands and face exposed was ineffective at the exposure times assessed. While the required sun exposure times for brown-skinned people are subject to several assumptions, it is nonetheless evident that sun exposure can usefully contribute to vitamin D status, even in the UK and for those with naturally pigmented skin, to help avoid deficiency in the summer months. However, to ensure deficiency is avoided year round, additional oral vitamin D for skin type V individuals is clearly pragmatic.

## Figures and Tables

**Table 1:** Model parameters assessed from previous in vivo research and outcome by exposure scenario S1–S4.

Model Parameters and Summary Results	
End summer month	August
End summer 25(OH)D target ^+^ (nmol/L)	85.8
Monthly 25(OH)D spend (nmol/L/month)	6.25
Summer dose required (SED)	89.6 *
Acceptable daily dose (SED)	2.75
Time for fixed daily dose (S1–3), (minutes)	25
Time range (S4) for daily dose of 2.75 SED at noon in June. Time (minutes) varies with latitude from southern England to northern Scotland	25–40
S1: 35% skin area March–September, maintains 25(OH)D status	Y
S2: 10% skin area March–May + September plus 35% skin area June–August, maintains 25(OH)D status	Y **
S3: 10% skin area all summer, maintains 25(OH)D status	N
S4: 35% skin area, June–August, D adjusted for latitude to give 2.75 SED, maintains 25(OH)D status	Y

^+^ Ensures 97.5% population remain ≥25 nmol/L 25(OH)D in February, and 50% will be ≥50 nmol/L [14]. * The dose is calculated as that on a horizontal surface, the adjustment for a vertical body has been made in calculation of the exposure received at the skin under a range of scenarios [15]. ** Easily achieved in southern England, marginal in northern Scotland. Y = Yes, N = No.

## Supplementary Materials

The following are available online at http://www.mdpi.com/2072-6643/10/4/457/s1, containing details of all underlying data.
