# Supplementary material for: Colour Counts: Sunlight and Skin Type as Drivers of Vitamin D Deficiency at UK Latitudes
Source: Nutrients. 2018 Apr 7;10(4):457. doi: 10.3390/nu10040457 (PMC5946242; doi:10.3390/nu10040457)
Supplement: Supplementary file 1 [file nutrients-10-00457-s001.docx]

**Webb et al. Supplementary Information**

Data Availability:

The underlying modelled climatology data and their validation against ground-based data have been previously published [reference 15]. The model, all model input data, and the data against which the model was validated are freely available online from international geophysical data centres.

The model itself is available at <http://www.libradtran.org>

The input data are available from:

<https://ozoneaq.gsfc.nasa.gov/data/toms/> (TOMS ozone)

<https://disc.sci.gsfc.nasa.gov/Aura/data-holdings/OMI> (OMI ozone)

<http://modis.gsfc.nasa.gov> (Cloud cover and optical depth; Aerosol optical depth)

<http://www.temis.nl/data/topo/dem2grid.html> (Digital elevation data)

The measured ground-based data used for validation are available at <http://www.woudc.org>

The full *in vivo* human data set is available from Professor L.E. Rhodes.

The data for circulating 25OHD by month in a white Caucasian adult population was previously published [reference 14, Figure 1], and for comparison in a skin type V population [reference 10].

The change in circulating 25OHD for skin type V adults after a 6 week course of simulated sunlight exposures has been previously published [references 7 and 11].
